# Oral Microbiota Community Dynamics Associated With Oral Squamous Cell Carcinoma Staging

**DOI:** 10.3389/fmicb.2018.00862

**Published:** 2018-05-03

**Authors:** Chia-Yu Yang, Yuan-Ming Yeh, Hai-Ying Yu, Chia-Yin Chin, Chia-Wei Hsu, Hsuan Liu, Po-Jung Huang, Song-Nian Hu, Chun-Ta Liao, Kai-Ping Chang, Yu-Liang Chang

**Affiliations:** ^1^Department of Microbiology and Immunology, College of Medicine, Chang Gung University, Taoyuan, Taiwan; ^2^Graduate Institute of Biomedical Sciences, College of Medicine, Chang Gung University, Taoyuan, Taiwan; ^3^Molecular Medicine Research Center, Chang Gung University, Taoyuan, Taiwan; ^4^Division of Colon and Rectal Surgery, Chang Gung Memorial Hospital, Linkou, Taiwan; ^5^Genomic Medicine Core Laboratory, Chang Gung Memorial Hospital, Linkou, Taiwan; ^6^CAS Key Laboratory of Genome Sciences and Information, Beijing Institute of Genomics, Chinese Academy of Sciences, Beijing, China; ^7^Department of Cell and Molecular Biology, College of Medicine, Chang Gung University, Taoyuan, Taiwan; ^8^Department of Biomedical Sciences, College of Medicine, Chang Gung University, Taoyuan, Taiwan; ^9^Department of Otolaryngology-Head and Neck Surgery, Chang Gung Memorial Hospital, Taoyuan, Taiwan; ^10^Department of Oral & Maxillofacial Surgery, Chang Gung Memorial Hospital, Taoyuan, Taiwan

**Keywords:** oral microbiome, complexity, community dysbiosis, cancer progression, 16S rRNA sequencing

## Abstract

Oral squamous cell carcinoma (OSCC) is a highly aggressive cancer and the fourth leading malignancy among males in Taiwan. Some pathogenic bacteria are associated with periodontitis and oral cancer. However, the comprehensive profile of the oral microbiome during the cancer's progression from the early stage to the late stage is still unclear. We profiled the oral microbiota and identified bacteria biomarkers associated with OSCC. The microbiota of an oral rinse from 51 healthy individuals and 197 OSCC patients at different stages were investigated using 16S rRNA V3V4 amplicon sequencing, followed by bioinformatics and statistical analyses. The oral microbiota communities from stage 4 patients showed significantly higher complexity than those from healthy controls. The populations also dynamically changed with the cancer's progression from stage 1 to stage 4. The predominant phyla in the oral samples showed variation in the relative abundance of *Fusobacteria, Bacteroidetes*, and *Actinobacteria*. The abundance of *Fusobacteria* increased significantly with the progression of oral cancer from the healthy controls (2.98%) to OSCC stage 1 (4.35%) through stage 4 (7.92%). At the genus level, the abundance of *Fusobacterium* increased, while the number of *Streptococcus, Haemophilus, Porphyromonas*, and *Actinomyces* decreased with cancer progression. *Fusobacterium periodonticum, Parvimonas micra, Streptococcus constellatus, Haemophilus influenza*, and *Filifactor alocis* were associated with OSCC, and they progressively increased in abundance from stage 1 to stage 4. The abundances of *Streptococcus mitis, Haemophilus parainfluenzae*, and *Porphyromonas pasteri* were inversely associated with OSCC progression. We selected a bacterial marker panel of three bacteria (upregulated *F. periodonticum*, down-regulated *S. mitis*, and *P. pasteri*), which had an AUC of 0.956 (95% CI = 0.925–0.986) in discriminating OSCC stage 4 from the healthy controls. Furthermore, the functional prediction of oral bacterial communities showed that genes involved in carbohydrate-related metabolism, such as methane metabolism, and energy-metabolism-related parameters, such as oxidative phosphorylation and carbon fixation in photosynthetic organisms, were enriched in late-stage OSCC, while those responsible for amino acid metabolism, such as folate biosynthesis and valine, leucine, and isoleucine biosynthesis, were significantly associated with the healthy controls. In conclusion, our results provided evidence of oral bacteria community changes during oral cancer progression and suggested the possibility of using bacteria as OSCC diagnostic markers.

## Introduction

Oral squamous cell carcinoma (OSCC) is a subset of head and neck squamous cell carcinoma (HNSCC) and accounts for more than 90% of all oral cancer Chinn and Myers (Chinn and Myers, 2015). The incidence of OSCC appears to be increasing worldwide, and this common cancer is the fourth most prevalent among males in Taiwan (Chen et al., 1999; Siegel et al., 2017). Despite advancements in surgical techniques, adjuvant radiotherapy, and chemotherapy, the overall 5-year survival rate of all OSCC patients is approximately 50–60%. Early detection and multidisciplinary treatment can increase the success rates of OSCC treatment (Haddad and Shin, 2008). Cigarette smoking, alcohol consumption, and betel quid chewing are major risk factors in oral cancers (Blot et al., 1988; Znaor et al., 2003; Lin et al., 2011). Other possible risk factors may include viral infection, fungal infection, and chronic periodontitis (Bagan et al., 2010; Rischin et al., 2015).

Chronic inflammation is reported to promote the development of various tumors, including OSCC (Feller et al., 2013). Poor periodontal health status, such as gingivitis and periodontitis, are other important risk factors in oral cancer (Tezal et al., 2009). Pathogenic bacterial colonization is highly correlated with inflammation and cancer progression (Wang and Ganly, 2014; Wang and Jia, 2016). Using bacterial culture, previous studies showed that the ratio of anaerobic to aerobic bacteria was increased on the surface of OSCC and *Porphyromonas gingivalis* and *Fusobacteria* were found to be more prevalent (Nagy et al., 1998; Bolz et al., 2014). Hopper et al. reported that various bacteria groups have been identified within OSCC tissues by bacterial culture (Hooper et al., 2006). However, it is well known that various oral bacteria cannot be cultured. Mager et al. reported high salivary counts of *C. gingivalis, P. melaninogenica*, and *Streptococcus mitis* in OSCC patients by screening the 40 most common oral bacteria using specific DNA probes (Mager et al., 2005).

Bacterial communities are commonly profiled by identifying the prokaryotic 16S ribosomal RNA gene (16S rRNA), which is approximately 1,500 bp long and contains nine variable regions interspersed between conserved regions (Kuczynski et al., 2011). The variable regions of bacteria 16S rRNA are frequently used in phylogenetic classifications to identify genera or species. It has been reported that 52 different phylotypes are present in OSCC using the partial sequencing of the 16S rRNA gene (Hooper et al., 2007). By using culture-independent 16S rRNA approaches and denaturing gradient gel electrophoresis (DGGE) fingerprints, Pushalkar et al. assessed the bacterial composition in OSCC tumor tissue (Pushalkar et al., 2012). Using 454 pyrosequencing, the microbial diversity in the saliva of OSCC was explored by Pushalkar et al. (2011). Schmidt et al. reported that the abundance of *Firmicutes* and *Actinobacteria* was significantly decreased compared to normal samples (Schmidt et al., 2014). Recently, many reports have used the 16S rRNA sequencing with a high-throughput sequencer for bacterial profiling in OSCC. AI-hebshi et al. showed that some specific species in tumor tissues, such as *Pseudomonas aeruginosa* and *Fusobacterium nucleatum*, are associated with OSCC (Al-Hebshi et al., 2017a). Another report indicated that five genera (*Bacillus, Enterococcus, Parvimonas, Peptostreptococcus*, and *Slackia*) showed differences between patients with epithelial precursor lesions and OSCC (Lee et al., 2017). Zhao et al. reported that a group of periodontitis-correlated taxa was enriched in OSCC tumor lesions (Zhao et al., 2017). However, there has been no report on the bacterial community composition in the oral cavity during the progression of OSCC from the early stage to the late stage, and the comprehensive bacteria profile in the Taiwanese population is unclear. Thus, the characterization of the microbiota changes associated with OSCC progression could help shed light on the mechanisms of diseases and identify useful biomarkers and therapeutic targets. In the present study, we collected 248 samples from healthy subjects and OSCC patients. We then constructed a 16S amplicon library and performed sequencing using the Illumina MiSeq platform. We hypothesized that the oral microbiota community could dynamically change in OSCC patients. We also investigated the functional profile of the oral microbiome through the phylogenetic reconstruction of unobserved states (PICRUSt) and revealed many pathways associated with OSCC.

## Materials and methods

### Study subjects and study design

In total, 248 oral rinse samples from patients were included in this cross-sectional study, which was approved by the Institutional Review Board at Chang Gung Memorial Hospital in Taiwan. Prior to the sample collection, written informed consent was obtained from all patients or their families. Patients were classified into four groups (Table 1): healthy (51 controls), OSCC stage 1 (41 patients), OSCC stages 2 and 3 (66 patients), and OSCC stage 4 (90 patients). The healthy controls were defined as individuals without any diagnosed diseases in the oral cavity. The OSCC stages were classified according to the AJCC staging manual (7th edition, 2010). Oral rinse samples were collected within 1–3 weeks after the disease diagnosis. All diagnoses of OSCC were confirmed by biopsy and pathological findings, and the diagnoses of healthy oral cavity were made after a thorough clinical examination. All participants were not undergoing antibiotics treatment at the time of sample collection. Patients rinsed their mouths with 30 ml of sterile normal saline for 30 s and spit into a 50-ml sterile tube. After collection, samples were centrifuged at 6,000 rpm for 30 min, and the cellular parts were collected and stored at −80°C until use.

**Table 1:** Clinical characteristics of the study population in this study.

**Characteristics**	**Healthy control**	**OSCC**		
		**Stage 1**	**Stage 2 and 3**	**Stage 4**
Number of patients	51	41	66	90
Age (years)				
Range	22–54	40–77	32–87	33–82
Mean ± SD	31.2 ± 8.6	53.7 ± 9.4	54.5 ± 11.9	52.3 ± 9.0
Gender				
Male	27 (52.9%)	35 (85.4%)	61 (92.4%)	81 (90.0%)
Female	24 (47.1%)	6 (14.6%)	5 (7.6%)	9 (10.0%)
T stage				
T1,2	–	41 (100.0%)	59 (89.4%)	13 (14.4%)
T3,4	–	0 (0.0%)	7 (10.6%)	77 (85.6%)
N stage				
N(-)	–	38 (92.7%)	53 (80.3%)	24 (26.7%)
N(+)	–	0 (0.0%)	11 (16.7%)	64 (71.1%)
NA	–	3 (7.3%)	2 (3.0%)	2 (2.2%)
Overall stage				
I	–	41 (100.0%)	–	–
II	–	–	49 (74.2%)	–
III	–	–	17 (25.8%)	–
IV	–	–	–	90 (100.0%)
Alcohol drinking				
NO	NA	18 (43.9%)	30 (45.5%)	39 (43.3%)
YES	NA	23 (56.1%)	36 (54.5%)	51 (56.7%)
Betel quid chewing				
NO	NA	21 (51.2%)	25 (37.9%)	29 (32.2%)
YES	NA	20 (48.8%)	41 (62.1%)	61 (67.8%)
Cigarette smoking				
NO	NA	15 (36.6%)	16 (24.2%)	27 (30.0%)
YES	NA	26 (63.4%)	50 (75.8%)	63 (70.0%)
Site				
Buccal mucosa	NA	7 (17.1%)	22 (33.3%)	28 (31.1%)
Tongue	NA	26 (63.4%)	19 (28.8%)	25 (27.8%)
Gingiva	NA	2 (4.9%)	9 (13.6%)	20 (22.2%)
Mouth floor	NA	3 (7.3%)	6 (9.1%)	2 (2.2%)
Others	NA	3 (7.3%)	10 (15.2%)	15 (16.7%)

### DNA extraction

The total genomic DNA of bacteria from oral rinse samples was isolated using the QIAamp DNA Microbiome Kit (Qiagen, USA). In brief, 500 μl of AHL buffer were added to 1 ml of sample for host cell lysis, followed by digestion of the host nucleic acids with 2.5 μl of benzonase and 20 μl of proteinase K. The host DNA was removed by centrifuging, and then, 200 μl of ATL buffer were added to the bacterial cells in a pathogen lysis tube L and vortexed using a TissueLyser LT for 10 min at 30 Hz. The bacterial DNA was washed twice, eluted using nuclease-free water, and stored at −80°C. The concentrations and qualities of the purified DNA were determined with a Qubit high-sensitivity dsDNA assay (Life Technologies).

### Oral microbiota profiling by 16S rRNA sequencing

An amplicon library was constructed from individual samples by PCR amplification targeting the 16S rRNA V3-V4 region (460 bp). Illumina adaptor overhang nucleotide sequences were added to these gene-specific sequences (16S amplicon PCR forward primer sequence = 5′-TCGTCGGCAGCGTCAGATGTGTATAAGAGACAG**CCTACGGGNGGCWGCAG**-3′, 16S amplicon PCR reverse primer sequence = 5′-GTCTCGTGGGCTCGGAGATGTGTATAAGAGACAG**GACTACHVGGGTATCTAATCC**-3′). The first PCR mixture contained 1 μM aliquots of both forward and reverse primers, 1X KAPA HiFi Hotstart Ready Mix, and bacteria genomic DNA (10 ng). The first PCR program was performed with 3 min of denaturation at 95°C; 25 cycles of denaturation at 95°C (30 s), annealing at 55°C (30 s), and extension at 72°C (30 s); and a final 72°C extension for 30 s. The first PCR products of the 16S V3-V4 amplicons were purified using AMPure XP beads, followed by index PCR. Each index PCR reaction mixture contained 5 μl of both Nextera XT index primer 1 and primer 2, 1X KAPA HiFi Hotstart Ready Mix, and the purified products of the first PCR experiment (5 μl). The index program was performed using 3 min of denaturation at 95°C; 8 cycles of denaturation at 95°C (30 s), annealing at 55°C (30 s), and extension at 72°C (30 s); and a final extension at 72°C for 5 min. The index PCR products were purified with AMPure XP beads. The final amplicon libraries were approximately 630 bp and were validated using an HT DNA High Sensitivity LabChip kit (Caliper, PerkinElmer, MA, USA). The multiplex amplified libraries were pooled equally with unique indices, and the pooled libraries were denatured with NaOH. Sequencing of the multiplexed pooled libraries was performed on a Miseq system with 2 × 300 paired-end v3 sequencing reagents (Illumina, USA).

### Bioinformatics analysis

The sequencing reads were initially demultiplexed using MiSeq Reporter v2.6 according to the sample barcodes. The resulting pairs of reads from each sample were merged to obtain longer reads (460 ± 50 bp) to improve taxonomy classification using FLASH (V1.2.11) (Magoc and Salzberg, 2011). Only samples with merged reads ≥100,000 were retained for subsequent analysis. Low-quality reads with *q*-value < 20 were filtered by the split_libraries_fastq.py script of QIIME (Version 1.9.1) (Caporaso et al., 2010). The package Cutadapt v1.14 was used to remove forward and reverse sequencing primers from the merged reads of each dataset. The resulting sequence tags were compared to the Gold database (http://drive5.com/uchime/gold.fa) to remove chimera sequences using the USEARCH package (http://www.drive5.com/uparse/) (Edgar, 2013). Only sequence tags with length >400 bp were retained for subsequent analysis. The operational taxonomic units (OTUs) were clustered at 97% sequence similarity using USEARCH and then assessed using BLASTN 2.6.0+ against four sets of 16S rRNA reference sequences, including HOMD RefSeq V14.5, HOMD RefSeq Extended V1.1, modified GreenGeneGold, and the NCBI's Microbial 16S set (Al-Hebshi et al., 2015, 2017b). Taxonomy classification was also assigned according to the Greengenes database. Clustal Omega software was used to construct phylogenetic trees from the representative sequences of the OTUs. The α-diversity (e.g., observed OTU numbers, Chao index, Simpson index, and Shannon index) and β-diversity (Bray Curtis dissimilarity) measurements were calculated based on the normalized data of the cumulative sum scaling (CSS) transformation of metagenomeSeq (Paulson et al., 2013). Canonical correspondence analysis (CCA) and constrained principal coordinate analysis (CPCoA) were used to visualize the data. The differential abundances of OTUs between healthy controls and OSCC were determined using Linear discriminant analysis Effect Size (LEfSe) (Segata et al., 2011). A heatmap was obtained using R scripts with the clustering distance of “correlation” and clustering method of the “ward.D2” settings. The metagenome content was predicted using PICRUSt (Langille et al., 2013), and the KEGG pathway was generated.

### Statistical analyses

The relative abundance of bacteria and alpha diversity indices were compared and displayed using GraphPad Prism 6 (GraphPad Software, Inc., La Jolla, CA, USA). Between-group comparisons were performed with a nonparametric Mann-Whitney U test for two groups. Receiver operator characteristic (ROC) curves were generated to illustrate the decision value of various bacteria biomarkers. The true positive rate (sensitivity) was plotted against the false positive rate (100% – specificity), and the area under the curve (AUC) values were reported with the 95% confidence interval as an estimate of diagnostic usefulness. We performed multivariate logistic regression analysis on the bacteria markers and used the statistical logistic forward method to produce a panel to identify OSCC stage 4 patients. Statistical analyses were performed using the SPSS software, version 12.0 (SPSS Inc., Chicago, IL, USA). The tests were two-sided, and a *P*-value < 0.05 was considered statistically significant.

## Results

### Subject characteristics and oral microbiota profiling by 16S rRNA sequencing

**Figure 1:**
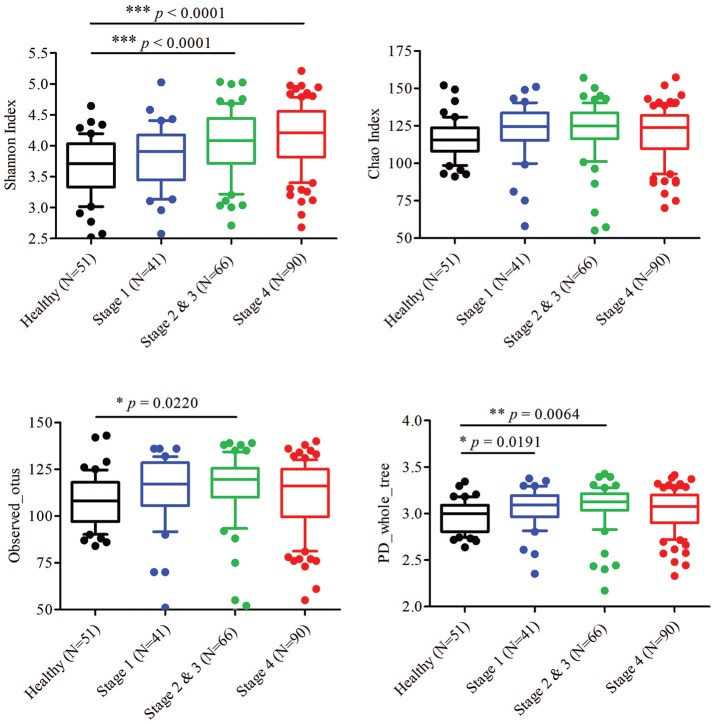
Diversity estimate calculations of bacterial taxa between healthy and OSCC patients. The Shannon index, Chao index, observed otus, and PD whole tree of the oral microbiome from healthy controls and OSCC patients. Horizontal lines represent mean values. *p* < 0.05 indicates statistical significance using a non-parametric Mann-Whitney U test.

**Figure 2:**
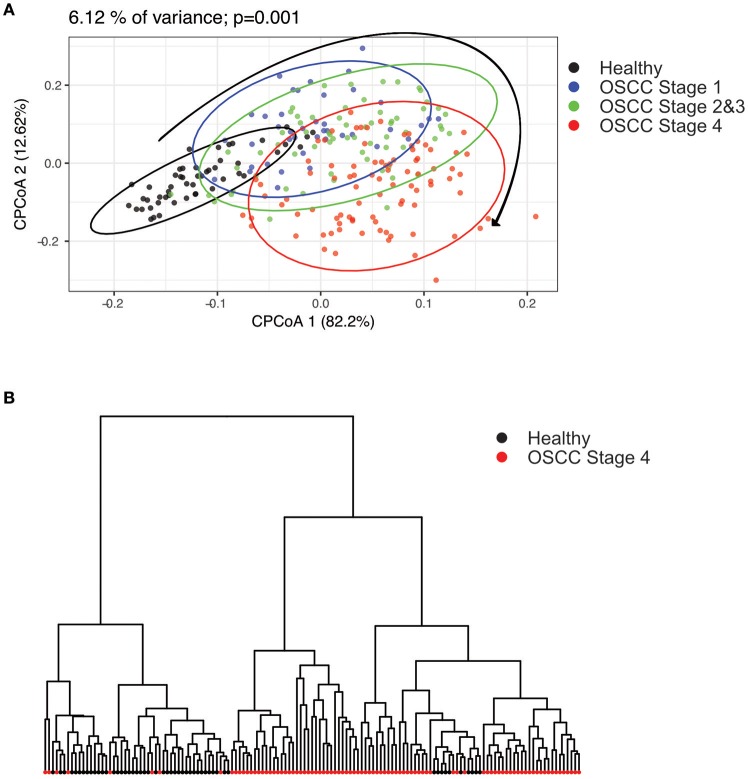
Principal component analysis (PCoA) and clustering analysis in healthy and OSCC patients. The oral microbiome compositions of healthy individuals and OSCC patients were analyzed using PCoA analysis and clustering analysis. **(A)** The individual samples are color coded to indicate healthy controls, stage 1, stages 2 and 3, and stage 4. The plot was analyzed and generated using the Bray-Curtis distances. **(B)** Hierarchical clustering was done using the healthy and OSCC stage 4 groups.

To characterize the oral microbiome community dynamics in OSCC, oral rinse specimens were collected from OSCC stage 1 patients (*N* = 41), stage 2 and 3 patients (*N* = 66), stage 4 patients (*N* = 90), and healthy controls (*N* = 51) (Table 1). The bacterial DNA was isolated from specimens, followed by PCR amplification targeting the 16S rRNA V3–V4 hypervariable region. The 16S amplicons were purified, and a second round of index PCR was performed. The multiplex amplified libraries were pooled equally and sequenced on an Illumina MiSeq system. The average number of raw paired reads per sample was 237,927 ± 140,766 reads for healthy controls, 214,765 ± 51,015 reads for OSCC stage 1 patients, 211,106 ± 36,167 reads for OSCC stage 2 and 3 patients, and 223,331 ± 61,191 reads for OSCC stage 4 patients (Supplementary Table 1). Two samples with a low number of combined paired reads (<100,000) were not included in the following analysis. After selecting the qualified reads, 33,090,959 quality-filtered reads were obtained from the 248 samples. The average numbers of quality-filtered reads per sample were 145,639 ± 121,079 reads for healthy controls, 124,747 ± 44,668 reads for OSCC stage 1 patients, 116,764 ± 30,104 reads for OSCC stage 2 and 3 patients, and 141,693 ± 54,096 reads for OSCC stage 4 patients (Supplementary Table 1). The clustering of the quality-filtered reads with a 98% sequence identity resulted in 424 unique OTUs in our oral microbiome datasets. A Venn diagram was used to show the shared or unique OTUs based on the OTUs present in 90% of the samples of a particular group. The Venn diagram displays that 89 OTUs were present in 90% of the subjects (Supplementary Figure 1). As shown in Supplementary Figure 1, 47 OTUs were common to all groups. Although these OTUs were present in most samples, it is important to note that there was a significant difference in their abundance across groups. There were 18 OTUs and 3 OTUs uniquely present in the healthy and OSCC stage 4 patients, respectively (Supplementary Figure 1). The oral microbiome complexity estimated in the rarefaction analysis indicated that the sequencing depth per sample covered most of the diversity and reached a saturated plateau phase (Supplementary Figure 2). The oral microbiota communities in late OSCC stages showed a significantly higher Shannon index (*p* < 0.0001) than the healthy controls (Figure 1). The OTU richness (Chao1 index, observed OTUs, PD whole tree) had a minor increased in OSCC patients compared to healthy controls (Figure 1). The beta diversity considers the difference in bacterial community composition for different groups. A scatter plot based on our principal coordinate analysis (PCoA) revealed that the oral microbiota in OSCC stage 4 patients was significantly different from those identified in the healthy controls (Figure 2A). More interestingly, we observed that the oral microbiota communities shifted progressively with the progression of oral cancer from the early stage to the late stage OSCC (Figure 2A). Hierarchical clustering analysis revealed that OSCC stage 4 patients formed a cluster and distinctly separated from the cluster of healthy controls (Figure 2B). These results suggested that the oral bacterial community was different between the healthy and OSCC groups and changed during the progression of OSCC.

### Differentially abundant phyla and genera in OSCC patients and healthy groups

**Figure 3:**
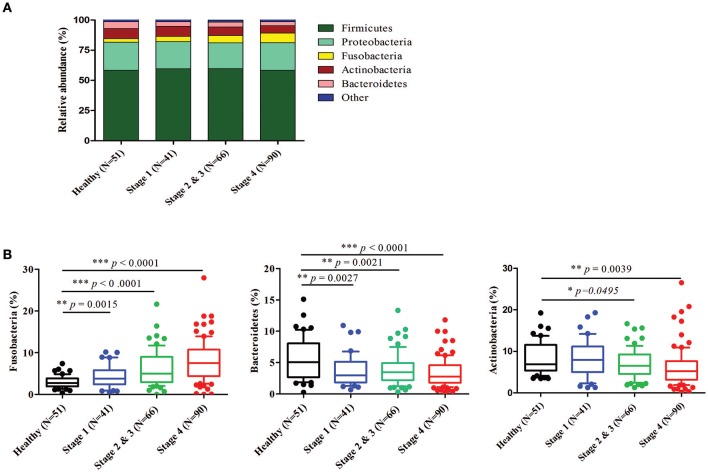
Relative abundance of different bacteria phyla in healthy and OSCC patients. Bacteria taxonomic profiling at the phylum level of oral microbiome from healthy controls and OSCC patients. **(A)** The five most abundant bacteria phylum in the oral microbiome. **(B)** Box plots show the relative abundance of *Fusobacteria, Bacteroidetes*, and *Actinobacteria* in healthy and OSCC patients. Horizontal lines represent mean values. *p* < 0.05 indicates statistical significance using a nonparametric Mann-Whitney U test.

**Figure 4:**
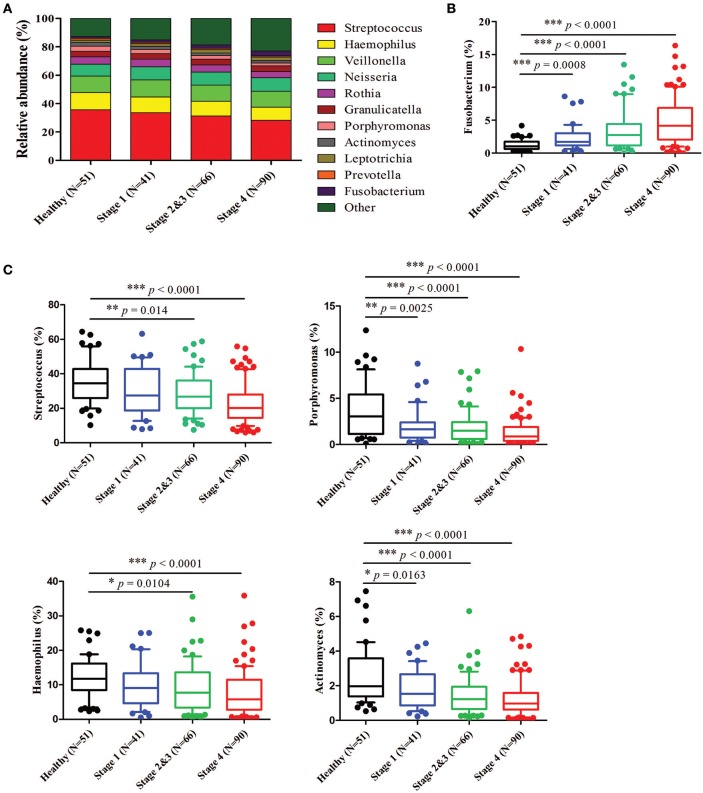
Relative abundance of the most prevalent genera in healthy and OSCC patients. Bacteria taxonomic profiling at the genus level of the oral microbiome from healthy controls and OSCC patients. **(A)** The 11 bacteria at the genus level in the oral microbiome. The box plots show the relative abundance of *Fusobacterium* **(B)** and *Streptococcus, Haemophilus, Porphyromonas*, and *Actinomyces* **(C)** in healthy controls and OSCC patients. Horizontal lines represent mean values. *p* < 0.05 indicates statistical significance using a nonparametric Mann-Whitney U test.

Further analysis of the relatively abundant 169 OTUs (frequency higher than 0.001) in our oral microbiota dataset revealed a total of nine phyla, of which five accounted for 99% of the bacteria. In the healthy controls, bacteria from *Firmicutes* (58.40%), followed by *Proteobacteria* (23.22%), *Actinobacteria* (8.36%), *Bacteroidetes* (5.65%), and *Fusobacteria* (2.98%) (Figure 3A) dominated. The alterations in the relative abundances of *Actinobacteria, Bacteroidetes*, and *Fusobacteria* were associated with tumor progression. Compared to healthy controls, the abundance of *Fusobacteria* was significantly increased with the progression of oral cancer from stage 1 (4.35%, *p* = 0.0015), stages 2 and 3 (6.24%, *p* < 0.0001), and then to stage 4 (7.92%, *p* < 0.0001). (Figure 3B). The amounts of *Bacteroidetes* and *Actinobacteria* also decreased significantly with the cancer progression from stage 1 to stage 4 (Figure 3B). The proportions of the other two phyla (*Firmicutes* and *Proteobacteria*) showed no significant changes (Supplementary Figure 3). According to the genus-level profiling, OTUs could be assigned to 53 individual genera, of which 11 were present in all samples with a relative abundance of more than 1% in at least one sample (Figure 4A, Supplementary Figure 4). The five predominant genera were *Streptococcus* (35.57%), *Haemophilus* (12.30%), *Veillonella* (11.56%), *Neisseria* (8.39%), and *Rothia* (5.12%) in the healthy controls. However, the ranking of the predominant bacteria was different in OSCC stage 4; the top five genera were *Streptococcus* (28.21%), *Veillonella* (11.01%), *Neisseria* (9.65%), *Haemophilus* (9.37%), and *Rothia* (4.42%) (Figure 4A). In the 11 most prevalent genera, the OSCC stage 4 samples showed 5 significantly different genera, with one genera increasing (*Fusobacterium*) (*p* < 0.0001) and 4 genera decreasing (*Streptococcus, Haemophilus, Porphyromonas*, and *Actinomyces*) (*p* < 0.0001) compared with the healthy controls (Figures 4B,C). Additionally, the relative abundances of these five genera were altered with cancer progression from stage 1 to stage 4. These results suggested that oral microbial dysbiosis was present in OSCC development.

### Differentially abundant OTUs in OSCC patients and healthy individuals

**Figure 5:**
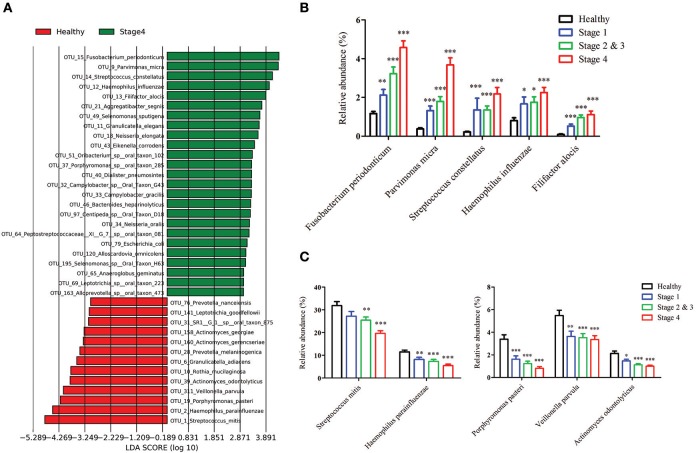
The differentially enriched bacteria in OSCC patients when compared to healthy controls. **(A)** Linear discriminant analysis effect size (LEfSe) analysis showing bacteria at the species level that were altered between the OSCC stage 4 and healthy controls. The relative abundance of the upregulated bacteria **(B)** and downregulated bacteria **(C)** at the species level in OSCC.

To identify the differentially enriched species within groups, the LEfSe method was used. *Fusobacterium periodonticum, Parvimonas micra, Streptococcus constellatus, Haemophilus influenza*, and *Filifactor alocis* were the most significantly abundant in the OSCC stage 4 samples, while *S. mitis, Haemophilus parainfluenzae, Porphyromonas pasteri, Veillonella parvula*, and *Actinomyces odontolyticus* were mostly associated with the healthy controls (Figure 5A). The relative abundances of these bacteria were progressively changed with cancer progression from stage 1 to stage 4 (Figures 5B,C). The efficacy of these differentially expressed bacteria in discriminating between OSCC stage 4 patients and healthy controls was calculated using a ROC curve. Among the 10 differentially expressed bacteria, the area under the curve (AUC) values for distinguishing OSCC stage 4 from healthy were 0.864 for *F. periodonticum*, 0.883 for *P. micra*, 0.856 for *S. constellatus*, and 0.857 for *P. pasteri* (Table 2). We selected a bacteria marker panel with three bacteria (upregulated *F. periodonticum* and down-regulated *S. mitis and P. pasteri)*, which had an AUC of 0.956 (95% CI = 0.925–0.986) in discriminating OSCC stage 4 from the healthy controls. Furthermore, we compared the differentially enriched species within stage 1 and healthy or stage 2 and 3 and healthy (Supplementary Figure 5). More importantly, 9 out of the 10 bacteria that were identified in stage 4 were also enriched in OSCC stage 1 to stage 3. These results collectively indicated that specific pathogenic bacteria showed significantly different expression in patients with OSCC stage 4 compared to the healthy controls. The abundance of these bacteria was associated with cancer stage. In addition, a useful bacterial panel to species level was generated from our oral microbiome datasets.

**Table 2:** The area under curves of potential bacteria biomarkers in discriminating OSCC stage 4 patients from healthy control.

**Bacteria biomarkers**	**Healthy**	**OSCC stage 4**	***p* value^b^**	**AUC (95% CI)**
**DOWN-REGULATED IN OSCC STAGE 4**				
*Streptococcus mitis* (OTU1)^a^	31.86 ± 1.75	19.65 ± 1.16	<0.001	0.780 (0.703–0.857)
*Haemophilus parainfluenzae* (OTU2)^a^	11.50 ± 0.80	5.45 ± 0.65	<0.001	0.805 (0.733–0.877)
*Porphyromonas pasteri* (OTU19)^a^	3.38 ± 0.39	0.82 ± 0.14	<0.001	0.857 (0.796–0.917)
*Veillonella parvula* (OTU311)^a^	5.47 ± 0.48	3.36 ± 0.34	<0.001	0.698 (0.611–0.785)
*Actinomyces odontolyticus* (OTU39)^a^	2.12 ± 0.21	1.00 ± 0.10	<0.001	0.778 (0.701–0.856)
**UP-REGULATED IN OSCC STAGE 4**				
*Fusobacterium periodonticum* (OTU15) ^a^	1.17 ± 0.11	4.58 ± 0.34	<0.001	0.864 (0.804–0.924)
*Parvimonas micra* (OTU9)^a^	0.38 ± 0.05	3.68 ± 0.36	<0.001	0.883 (0.827–0.940)
*Streptococcus constellatus* (OTU14)^a^	0.22 ± 0.03	2.18 ± 0.34	<0.001	0.856 (0.792–0.921)
*Haemophilus influenza* (OTU12)^a^	0.80 ± 0.14	2.25 ± 0.26	<0.001	0.696 (0.609–0.782)
*Filifactor alocis* (OTU13)^a^	0.09 ± 0.03	1.12 ± 0.18	<0.001	0.800 (0.728–0.872)

a *Data are presented as mean ± s.e.m*.

b *The p-value of Mann-Whitney U test presents the difference between healthy control and OSCC stage 4*.

### Association between oral bacteria biomarkers and clinical parameters

Next, we analyzed the differentially expressed bacteria with various clinicopathologic manifestations. Among the five upregulated bacteria, the abundances of three bacteria (*F. periodonticum, P. micra*, and *S. constellatus*) were significantly increased with the T stage and overall pathologic stage (*p* ≤ 0.001, Table 3). The levels of *F. periodonticum* and *P. micra* significantly increased with the N stage (*p* < 0.01, Table 3). Interestingly, the amount of *F. alocis* was significantly increased in OSCC patients who were smokers (*p* < 0.01, Table 3). Among the five down-regulated bacteria, the levels of four bacteria (*S. mitis, H. parainfluenzae, P. pasteri*, and *A. odontolyticus*) were significantly decreased with the T stage and the overall pathologic stage (*p* < 0.05, Table 4). The abundance of *H. parainfluenzae* was significantly decreased with the N stage (*p* < 0.05, Table 4).

**Table 3:** Clinicopathological characteristics related to the increased five bacteria biomarkers in OSCC.

**Characteristics**	**Patients numbers**	***Fusobacterium periodonticum*^a^**	***p*-value^b^**	***Parvimonas micra*^a^**	***p-*value^b^**	***Streptococcus constellatus*^a^**	***p*-value^b^**	***Haemophilus influenza*^a^**	***p-*value^b^**	***Filifactor alocis*^a^**	***p* value^b^**
**AGE**											
>52 years	93	3.96 ± 0.34	0.340	2.47 ± 0.28	0.926	1.67 ± 0.32	0.863	2.17 ± 0.27	0.246	0.92 ± 0.16	0.295
<52 years	104	3.30 ± 0.26		2.63 ± 0.30		1.79 ± 0.27		1.77 ± 0.21		0.96 ± 0.12	
**GENDER**											
Male	117	3.66 ± 0.22	0.142	2.58 ± 0.22	0.399	1.58 ± 0.18	0.634	2.01 ± 0.18	0.284	0.99 ± 0.11	0.012
Female	20	3.15 ± 0.79		2.30 ± 0.57		3.08 ± 1.35		1.49 ± 0.41		0.46 ± 0.18	
**pT STATUS**											
I-II	113	2.89 ± 0.24	0.000	1.58 ± 0.17	0.000	1.26 ± 0.25	0.000	1.84 ± 0.21	0.760	0.81 ± 0.11	0.312
III-IV	84	4.59 ± 0.35		3.87 ± 0.38		2.36 ± 0.35		2.12 ± 0.28		1.11 ± 0.18	
**pN STATUS**											
0	115	3.08 ± 0.25	0.001	1.95 ± 0.19	0.002	1.45 ± 0.24	0.118	2.08 ± 0.24	0.628	0.77 ± 0.09	0.433
>0	75	4.37 ± 0.36		3.52 ± 0.42		2.00 ± 0.36		1.91 ± 0.24		1.07 ± 0.18	
**OVERALL STAGE**											
I-II	90	2.75 ± 0.26	0.000	1.42 ± 0.15	0.000	1.31 ± 0.30	0.001	1.86 ± 0.25	0.567	0.69 ± 0.09	0.068
III-IV	107	4.34 ± 0.31		3.51 ± 0.33		2.08 ± 0.29		2.05 ± 0.23		1.15 ± 0.16	
**SMOKING**											
Yes	139	3.51 ± 0.25	0.284	2.45 ± 0.23	0.641	1.64 ± 0.22	0.893	1.99 ± 0.21	0.635	1.09 ± 0.13	0.006
No	58	3.86 ± 0.39		2.80 ± 0.42		1.94 ± 0.47		1.89 ± 0.28		0.57 ± 0.10	
**ALCOHOL**											
Yes	110	3.44 ± 0.25	0.768	2.51 ± 0.26	0.704	1.91 ± 0.32	0.319	1.96 ± 0.24	0.559	0.97 ± 0.14	0.166
No	87	3.82 ± 0.36		2.61 ± 0.32		1.50 ± 0.25		1.97 ± 0.24		0.90 ± 0.14	

a *Data are presented as mean ± s.e.m*.

b *The p-value is calculated using Mann-Whitney U test*.

**Table 4:** Clinicopathological characteristics related to the decreased five bacteria biomarkers in OSCC.

**Characteristics**	**Patients numbers**	***Streptococcus mitis*^a^**	***p* value^b^**	***Haemophilus parainfluenzae*^a^**	***p* value^b^**	***Porphyromonas pasteri*^a^**	***p* value^b^**	***Veillonella parvula*^a^**	***p* value^b^**	***Actinomyces odontolyticus*^a^**	***p* value^b^**
**AGE**											
>52 years	93	21.63 ± 1.25	0.046	6.39 ± 0.62	0.966	1.05 ± 0.15	0.875	3.70 ± 0.35	0.546	1.05 ± 0.08	0.538
<52 years	104	24.54 ± 1.17		6.87 ± 0.68		1.18 ± 0.18		3.26 ± 0.27		1.21 ± 0.10	
**GENDER**											
Male	177	23.07 ± 0.92	0.605	6.48 ± 0.46	0.869	1.11 ± 0.13	0.424	3.44 ± 0.23	0.661	1.14 ± 0.07	0.987
Female	20	24.00 ± 2.38		8.06 ± 2.08		1.19 ± 0.30		3.72 ± 0.69		1.14 ± 0.22	
**pT STATUS**											
I–II	113	25.69 ± 1.14	0.000	7.75 ± 0.62	0.000	1.39 ± 0.17	0.000	3.65 ± 0.28	0.171	1.24 ± 0.09	0.017
III–IV	84	19.77 ± 1.22		5.16 ± 0.65		0.75 ± 0.15		3.22 ± 0.34		1.00 ± 0.10	
**pN STATUS**											
0	115	24.52 ± 1.08	0.054	7.18 ± 0.62	0.026	1.32 ± 0.17	0.075	3.58 ± 0.29	0.761	1.14 ± 0.08	0.877
>0	75	21.64 ± 1.42		5.53 ± 0.67		0.90 ± 0.17		3.50 ± 0.35		1.08 ± 0.10	
**OVERALL STAGE**											
I-II	90	25.88 ± 1.25	0.001	7.60 ± 0.68	0.006	1.50 ± 0.20	0.002	3.52 ± 0.31	0.619	1.01 ± 0.09	0.031
III-IV	107	20.88 ± 1.14		5.84 ± 0.62		0.80 ± 0.13		3.43 ± 0.30		1.28 ± 0.10	
**SMOKING**											
Yes	139	24.39 ± 1.04	0.025	6.34 ± 0.56	0.132	1.04 ± 0.13	0.141	3.41 ± 0.27	0.409	1.09 ± 0.08	0.213
No	58	20.23 ± 1.46		7.36 ± 0.80		1.32 ± 0.23		3.62 ± 0.37		1.23 ± 0.12	
**ALCOHOL**											
Yes	110	24.15 ± 1.14	0.131	6.20 ± 0.65	0.052	1.00 ± 0.14	0.432	3.24 ± 0.26	0.328	1.10 ± 0.09	0.212
No	87	21.93 ± 1.30		7.21 ± 0.64		1.28 ± 0.20		3.77 ± 0.36		1.18 ± 0.10	

a *Data are presented as mean ± s.e.m*.

b *The p-value is calculated using Mann-Whitney U test*.

### Predicated oral microbiome function using picrust

**Figure 6:**
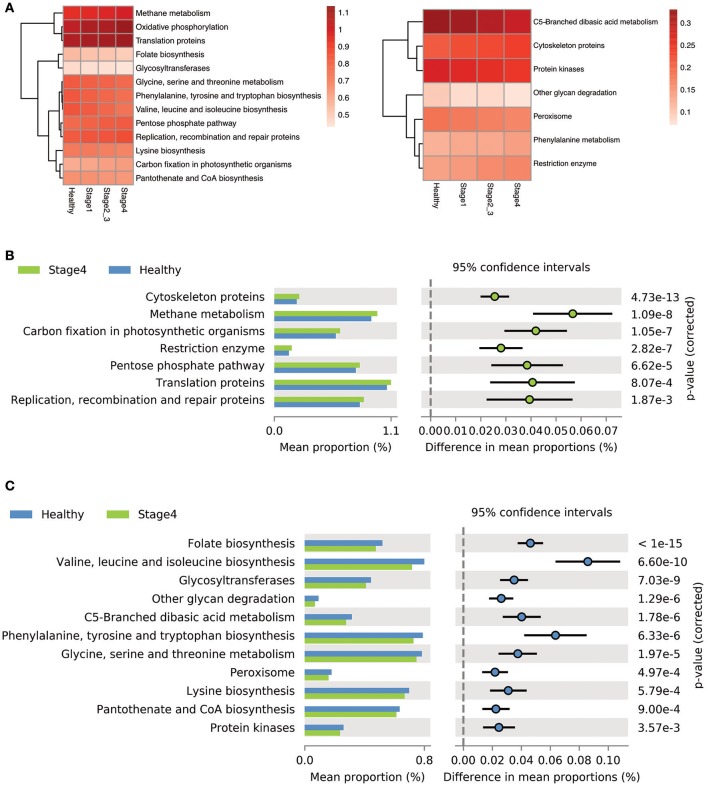
Differentially enriched functions between healthy controls and OSCC patients by PICRUSt analysis. **(A)** Comparison of the relative abundance of the PICRUSt-generated functional profile of the saliva microbiome in OSCC patients and healthy controls. **(B)** The upregulated pathways in OSCC stage 4 patients. **(C)** The down-regulated pathways in OSCC stage 4 patients.

We used the Phylogenetic Investigation of Communities by Reconstruction of Unobserved States (PICRUSt) to predict the oral microbiome functions in the OSCC and healthy saliva microbiome datasets. Carbohydrate-related metabolism, such as methane metabolism, and the levels of energy-metabolism-related parameters, such as oxidative phosphorylation and carbon fixation in photosynthetic organisms, were associated with the progression of OSCC and were related to the bacterial abundance changes from stage 1 to stage 4 (Figures 6A,B, Supplementary Figure 6). In contrast, parameters related to protein and amino acid metabolism, such as valine, leucine and isoleucine, phenylalanine, tyrosine, and tryptophan biosynthesis, and folate biosynthesis, were inversely associated with OSCC progression, as their abundance decreased in abundance from stage 1 to stage 4 (Figures 6A,C, Supplementary Figure 6).

## Discussion

Various studies have reported that many pathogenic bacteria are found in periodontitis patients or oral cancer patients compared to healthy controls. Since most of these studies were not focused on OSCC patients with different stages, we hypothesized that cancer progression may lead to different oral microbiome. In this study, we provided a comprehensive profile of the oral microbiota community dynamics of OSCC patients in different stages. Using 16S rRNA sequencing analysis to profile the oral microbiome, we found that the Shannon index increased for both the richness and the evenness of the community in late stage patients compared with healthy controls. Interestingly, the bacterial community composition changed dynamically with the progression of oral cancer and clearly showed different populations in the PCA plot between the healthy controls and OSCC stage 4 patients. These findings suggest that the oral microbiome community experiences imbalance during the progression of cancer.

In our bacteriome datasets, five major bacteria phyla accounting for 99% of the bacteria were identified in the samples from healthy and OSCC groups. *Firmicutes* was the most dominant phylum in the oral rinse samples; the relative abundances of *Firmicutes* were approximately 58.40% in healthy individuals, 59.65% in OSCC stage 1 patients, 59.76% in OSCC stage 2 and 3 patients, and 58.43% in OSCC stage 4 patients. It has been reported that the relative abundance of *Firmicutes* is approximately 25% in the tumor lesions, while the abundance of *Firmicutes* was approximately 35% in the saliva in OSCC patients. The oral rinse samples contain a mixture of bacteria released from numerous diverse microenvironments in the oral cavity; thus, they may not fully present the microbiome specifically associated with the tumor lesions. Additionally, different sampling methods for saliva and oral rinse may enrich specific bacteria. Among the phyla identified, OSCC stage 4 patients had significantly more *Fusobacteria* than healthy individuals. *Fusobacteria* was the fifth most dominant bacteria in healthy controls, while it was the third most dominant in OSCC stage 4 patients. Additionally, our results indicate that the abundance of *Fusobacteria* was elevated in OSCC patients during the progression of cancer.

Many taxa were found to be differentially abundant between OSCC and healthy controls using LEfSe. At the species level, we identified five bacteria (*F. periodonticum, P. micra, S. constellatus, H. influenza*, and *F. alocis*) were the most significantly overrepresented in OSCC patients and the relative abundance of these bacteria were increased with cancer progression. Recent reports identified that OSCC patients were characterized by a significant abundance of several members of *Fusobacteriem* in the tumor lesions (Al-Hebshi et al., 2017a; Zhao et al., 2017). Consistently, many reports identified *Fusobacterium* at significantly higher levels in OSCC tumor tissues compared to normal tissue (Nagy et al., 1998; Schmidt et al., 2014). In this study, we explored the significance of *F. periodonticum* in the progression of oral cancer, which was increased from stage 1 (1.66%) to stages 2 and 3 (2.41%) and then to stage 4 (3.31%) in oral rinse samples from OSCC patients. Mager et al. detected *F. periodonticum* in the saliva sample from OSCC patients using specific bacteria probes, but its abundance showed no significantly difference between OSCC-positive and OSCC-free patients (2013). Another report indicated that *F. nucleatum* was the most significantly overexpressed species in tumors compared with control tissues by sequencing 16S rRNA V1-V3 region (Al-Hebshi et al., 2017a). We also detected *F. nucleatum* in our bacteriome datasets including healthy controls and OSCC, but its abundance showed no significant difference. This result may be due to different types of specimens collected or the different ethnic background and lifestyle of our subjects. *Parvimonas* is a Gram-positive anaerobic coccus that is a known oral pathogen and is associated with periodontitis in humans (Al-Hebshi et al., 2014). Another report indicated that *Parvimonas* is also enriched in tumor lesions (Lee et al., 2017; Zhao et al., 2017). *P. micra* was also reported to be enriched in OSCC tumor lesions by Al-Hebshi et al. (2017a). We found that the amount of *P. micra* in oral rinse sample was 0.38% in healthy controls, but it significantly increased to 1.31% in OSCC stage 1 patients, 1.72% in OSCC stage 2 and 3 patients, and 3.68% in OSCC stage 4 patients. Collectively, our results indicated that *P. micra* was also detected in oral rinse samples and may be associated with tumor stages. *Filifactor* is Gram-positive, slow-growing, and an obligate anaerobic bacteria from the phylum *Firmicutes*. Several recent studies reported that the amount of *F. alocis* was increased at sites of periodontal disease compared to healthy sites within the oral cavity and may be a new emerging periodontal pathogen (Schlafer et al., 2010; Aruni et al., 2015). It has been reported that *F. alocis* has synergistic interactions with other common periodontal bacteria, which leads to the colonization of pathogenic periodontal communities and cancer progression (Aruni et al., 2014). For the first time, we found that the amount of *F. alocis* was lower than 0.1% in healthy controls, but it was significantly increased by approximately 10-fold in OSCC stage 4 patients (1.12%). Furthermore, among the OSCC patients, the relative abundance of *F. alocis* was significantly increased and associated with smoking. *S. constellatus* are normal flora in oral cavities and upper respiratory tracts. It has been found to be involved with pulmonary exacerbations in cystic fibrosis patients (Sibley et al., 2008). For the first time, we explored the significant overexpression of *S. constellatus* in OSCC patients. In our study, *S. mitis* and *Haemophilus parainfluenza* were among the top taxa associated with healthy controls, which is consistent with findings from other previous studies using oral tissues (Pushalkar et al., 2011; Al-Hebshi et al., 2017a). In contradiction, Mager et al. showed that *S. mitis* was more abundant in saliva samples from OSCC patients (Mager et al., 2005). *P. pasteri* is a gram-negative, anaerobic bacterium that has been isolated from the human saliva. In this study, we are the first to report that the abundance of *P. pasteri* was decreased in OSCC patients compared to healthy individuals. Additionally, the amount of *P. pasteri* may be a useful bacteria marker for OSCC diagnosis.

In our microbiome datasets, no unique OTUs were observed in early stage patients, but 3 unique OTUs were present in OSCC stage 4 patients. Additionally, there were 18 unique OTUs in the healthy controls. One of the unique OTUs in stage 4 was the genus *Tannerella*, which is a Gram-negative anaerobic bacterium. *T. forsythia* was previously reported as a periodontal pathogen in this genus (Rylev and Kilian, 2008) These data suggest that the microenvironment of the mouth in late-stage oral cancer patients may facilitate the growth of some pathogenic species, such as those from *Tannerella*. Collectively, we explored the significance of oral pathogens in the progression of oral cancer, not only in association with periodontal disease in humans.

To determine stage-specific bacteria of OSCC, OTUs with different abundances in each stage of OSCC have been identified using LDA analysis. As shown in Figure 5A and Supplementary Figure 5, we identified potential stage specific bacteria, including *Neisseria elongata, Eikenella corrodens, Oribacterium sp._oral_taxon_102*, and *Dialister pneumosintes*, which were exclusively enriched in OSCC stage 4. Furthermore, the relative amounts of five OTUs (*F. periodonticum, P. micra, S. constellatus, H. influenza*, and *F. alocis*) were elevated in all stages of OSCC and positively associated with the stages of OSCC (Figure 5B), suggesting that the five OTUs could be used for early detection and/or monitoring the progression of OSCC. We also analyzed bacterial marker panels for early stages. However, we can't get a marker panel, which had an AUC higher than 0.90, for early stage OSCC with only three bacterial species or less. A bacterial marker panel with seven bacteria (upregulated *P. micra, S. constellatus, F. alocis* and downregulated *S. mitis, H. parainfluenzae, V. parvula, A. odontolyticus*) had an AUC of 0.894 in discriminating between stage 1 from healthy. Also, a bacterial marker panel with six bacteria (upregulated *F. periodonticum* and *P. micra* and downregulated *S. mitis, V. parvula, P. pasteri, A. odontolyticus*) had an AUC of 0.949 in discriminating between stage 2 and 3 from healthy. Functional prediction showed carbohydrate-related metabolism, such as methane metabolism, was significantly higher in OSCC stage 4 patients. Some bacteria were capable of producing energy through the reduction of CO_2_ to methane. Methane production has been shown to be associated with intestinal diseases, such as inflammatory bowel diseases and colon cancer (Roccarina et al., 2010; Zamani et al., 2014). We also explored the association of methane metabolism and OSCC. Furthermore, the levels of energy-metabolism-related parameters, such as oxidative phosphorylation and carbon fixation in photosynthetic organisms, were significantly higher in OSCC stage 4 patients than in the healthy controls. The pathways of oxidative phosphorylation and carbon fixation in photosynthetic organisms were reported to be associated with many inflammatory diseases and cancer (Jezierska-Drutel et al., 2013).

The present study has some limitations. The major limitations of the current study were that the samples derived from the healthy controls were not matched to the disease group with respect to age, gender and oral health status. Additionally, the overall oral health parameters of the participants were unidentified. Second, the different eating behaviors or hygiene habits of healthy and OSCC patients may affect the bacterial colony properties, which would have influenced the outcomes. The third limitation was that oral rinse samples may identify the specific bacteria that were not fully present in the tumor lesion. The different DNA extraction methods in various reports may result in differentially enriched taxa in OSCC. To our knowledge, this is the first clinical, comprehensive 16S rRNA sequencing dataset to characterize the community dynamics of oral microbiota in OSCC with different stages from the early stage to the late stage using next-generation sequencing technology. This high-throughput sequencing approach gives us the opportunity to discover new bacteria biomarkers that have not been previously reported for OSCC. In conclusion, the results reveal that the oral microbiota community dynamically changes during the progression of oral cancer and a bacteria marker panel (upregulated *F. periodonticum* and down-regulated *S. mitis* and *P. pasteri*) can discriminate OSCC stage 4 patients from the healthy controls.

## Author contributions

C-YY performed the study design, data analysis, drafting, revising, and final approval, and handled the accountability of all aspects of the work. Y-MY and H-YY performed the bioinformatics analysis and data acquisition. C-YC, C-WH, HL, P-JH, S-NH, and C-TL performed the data analysis and acquisition. Y-LC and K-PC performed the study design, clinical sample collection, data analysis, drafting, revising, and final approval and were also involved in the accountability of all aspects of the work. All authors had approved the final version of the work.

### Conflict of interest statement

The authors declare that the research was conducted in the absence of any commercial or financial relationships that could be construed as a potential conflict of interest.
