# Supplementary material for: Oral Microbiota Community Dynamics Associated With Oral Squamous Cell Carcinoma Staging
Source: Front Microbiol. 2018 May 3;9:862. doi: 10.3389/fmicb.2018.00862 (PMC5943489; doi:10.3389/fmicb.2018.00862)
Supplement: Supplementary file 1 [file Presentation_1.pdf]

Supplementary material
Figures

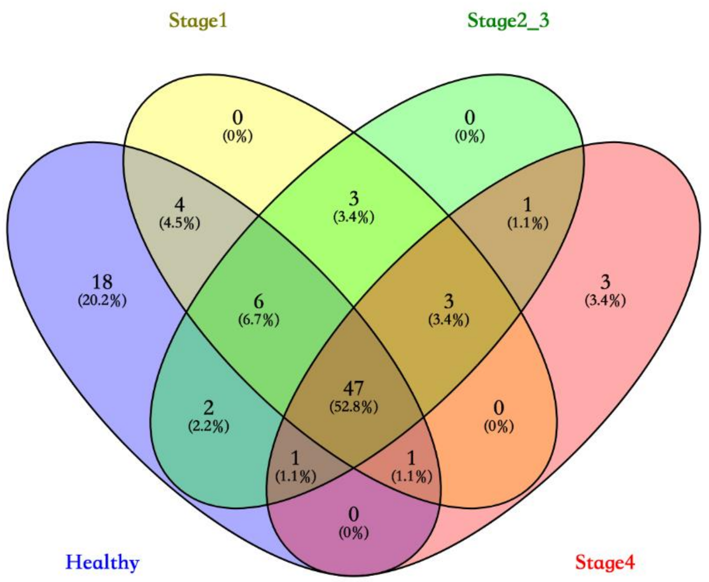

Supplementary Fig 1. Shared and unique OTUs in the oral microbiome in healthy and OSCC patients. Venn diagram displays the number of shared and unique OTUs based on the 89 OTUs present in 90% of the samples in the particular group including healthy, OSCC stage 1, OSCC stage 2&3, and OSCC stage 4.

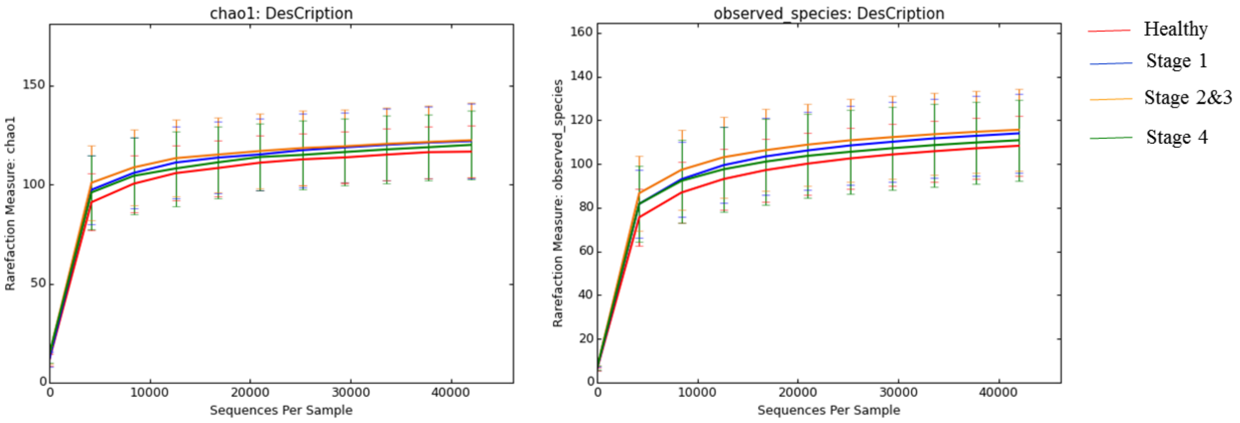

Supplementary Fig 2. Alpha diversity analysis of oral microbiome in healthy and OSCC patients. Rarefaction analysis of the Chao1 index and observed species were performed and different colors represented different samples: healthy (red), stage 1 (blue), stage 2&3 (orange), and stage 4 (green). Mean ± SEM were shown.

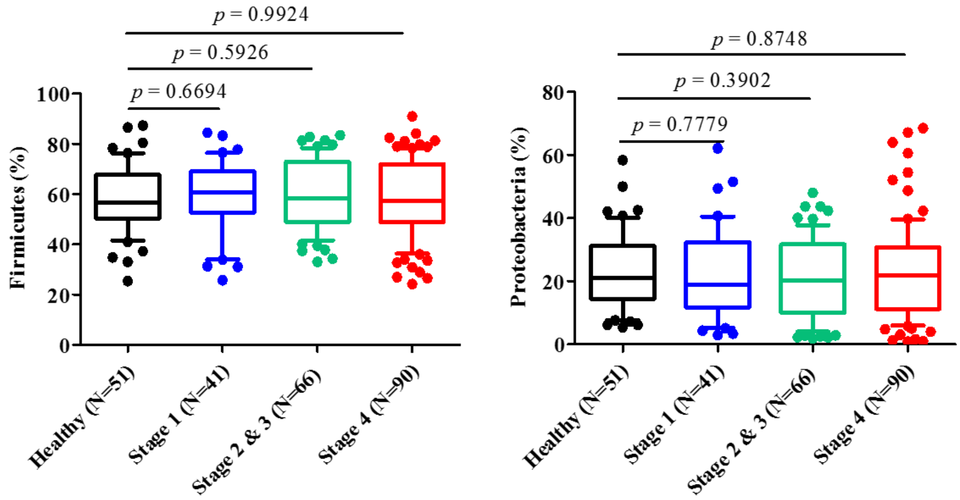

Supplementary Fig 3. Relative abundance of the *Firmicutes* and *Proteobacteria* in healthy and OSCC patients. The box plot represented the relative abundance of bacteria phylum in healthy control and OSCC patients. Horizontal lines represented mean values. The p value was calculated by nonparametric Mann-Whitney U test. A p value < 0.05 indicated the statistical significance.

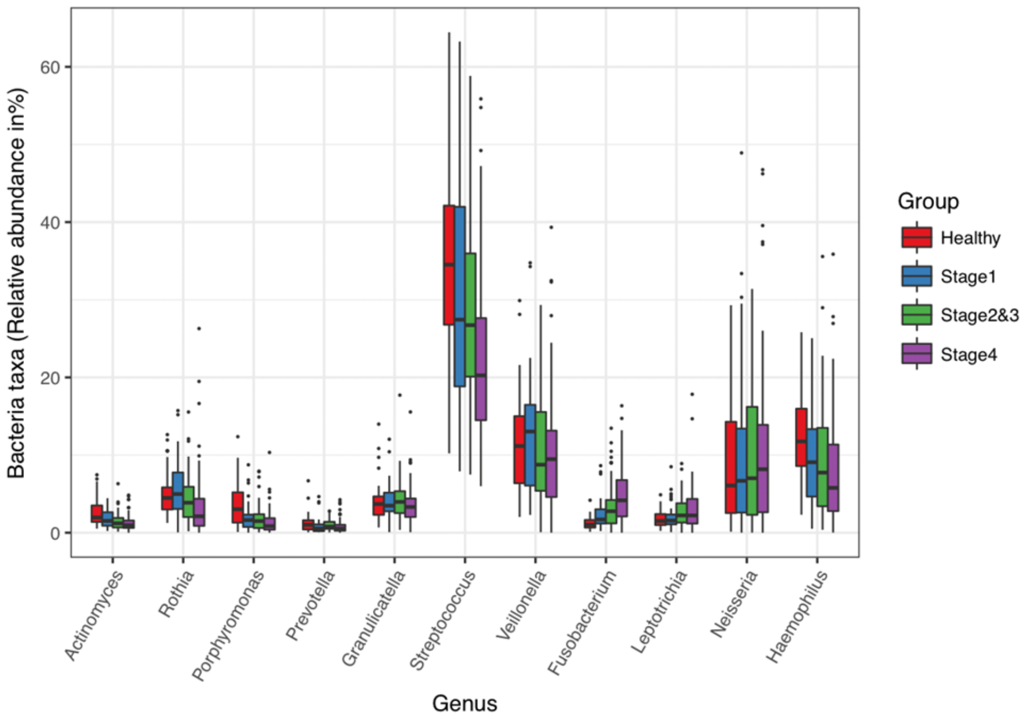

Supplementary Fig 4. The most predominant 11 genera in healthy and OSCC patients. The bar showed the 11 most abundance bacteria at genus level in oral microbiome. Horizontal lines represented mean values.

(A)

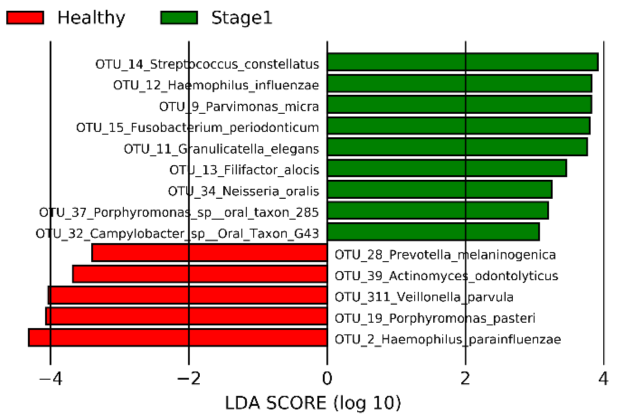

(B)

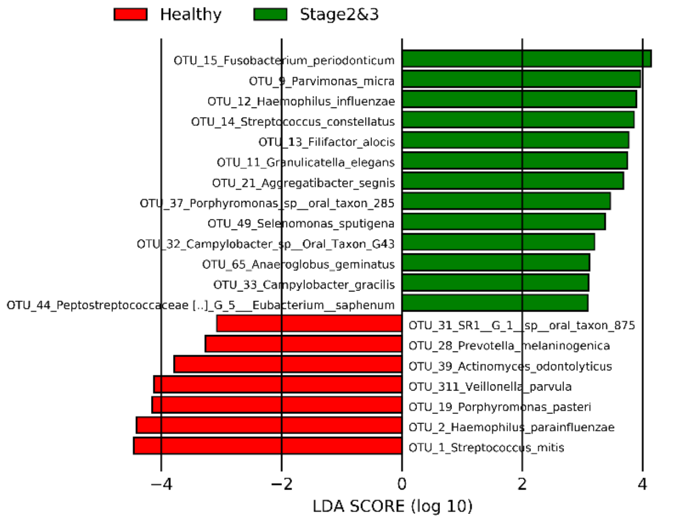

Supplementary Fig 5. Distinct taxa identified in the OSCC and healthy using LefSe analysis. LDA acores showed significant bacterial differentces within OSCC stage 1 and healthy (A) and OSCC stage2&3 and healthy (B).

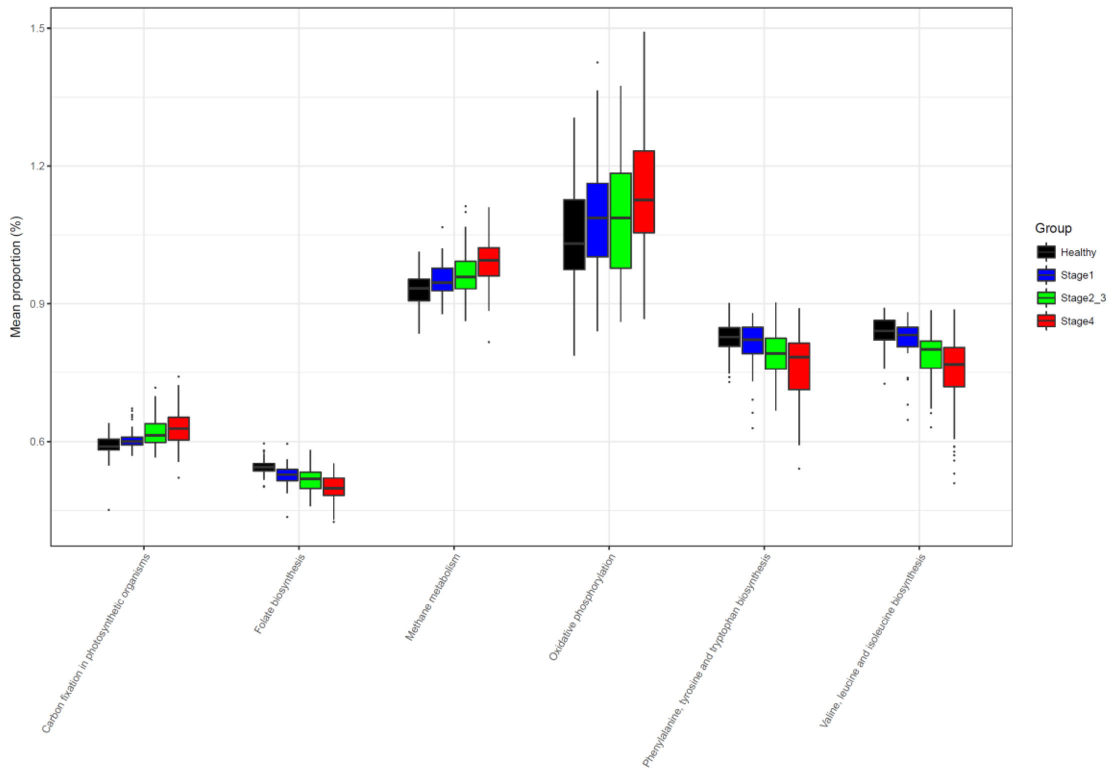

Supplementary Fig 6. The relative abundance of the PICRUSt-generated functional profile of the saliva microbiome in OSCC patients and healthy controls.

Tables

Supplementary Table 1. Summary of the sequence statistics.

	Healthy control	OSCC		
		Stage 1	Stage 2 and 3	Stage 4
Number of patients	51	41	66	90
Raw paired reads (per sample)				
Range	133,036 ~ 1,009,651	159,591 ~ 441,291	133,792 ~ 324,839	134,098 ~ 586,612
mean ± SD	237,927 ± 140,766	214,765 ± 51,015	211,106 ± 36,167	223,331 ± 61,191
Combined paired reads (per sample)				
Range	102,145 ~ 896,199	116,722 ~ 392,335	107,556 ~ 229,248	102,592 ~ 529,908
mean ± SD	190,523 ± 127,853	168,638 ± 42,576	162,323 ± 25,623	177,002 ± 57,067
Quality-filtered reads (per sample)				
Range	65,831 ~ 805,136	57,585 ~ 351,212	55,094 ~ 186,471	76,644 ~ 472,897
mean ± SD	145,639 ± 121,079	124,747 ± 44,668	116,764 ± 30,104	142,693 ± 54,096
